# Evaluating long-term care insurance policies in China: an integrated analysis of policy instruments and PMC index model

**DOI:** 10.3389/fpubh.2025.1740854

**Published:** 2026-01-13

**Authors:** Jin Zhou, Xuefang Yao, Lei Tian

**Affiliations:** 1 School of Health Economics and Management, Nanjing University of Chinese Medicine, Nanjing, Jiangsu, China; 2 International Pharmaceutical Business School, China Pharmaceutical University, Nanjing, Jiangsu, China

**Keywords:** long-term care, long-term care insurance, PMC index model, policy instrument, policy scoring

## Abstract

**Introduction:**

Widespread population aging has significantly intensified the pressure on caring for older adults with disabilities. Establishing a long-term care insurance (LTCI) system has emerged as a pivotal strategy for many countries to mitigate this challenge. China is currently in a critical phase of implementing its LTCI system, and a systematic evaluation of its policy instrument is needed to optimize the design and implementation thereof. This study aimed to establish a two-dimensional analytical “policy instrument–policy level” framework to conduct a qualitative analysis of LTCI policy texts from the promotion phase.

**Methods:**

We established a two-dimensional analytical framework. By integrating the Policy Modeling Consistency (PMC) index model, we selected a total of 45 LTCI policy documents issued between January 1, 2020, and April 1, 2025, for qualitative analysis and policy scoring. Eleven documents were issued at the central level by national institutions, and 34 at the local level by provincial or municipal government departments.

**Results:**

Environment-based policy instruments accounted for the predominant proportion (48.9%) among all instruments utilized, whereas supply-based (27.8%) and demand-based instruments (23.3%) were relatively underutilized. The central level employed policy instruments in a comparatively balanced manner, while the local level demonstrated a pronounced reliance on environment-based instruments (51.0%). Furthermore, the PMC index evaluation indicated that the average PMC index for the 45 policies was 6.01, which corresponds to an Acceptable average policy grade. Areas for improvement were identified, specifically concerning policy targets, policy timeframe, and policy level coordination.

**Conclusion:**

China’s LTCI policy framework demonstrates an acceptable overall performance level. However, it is characterized by a structural imbalance in the use of policy instruments, differing emphases between the central and local levels, and inadequate systematic design in certain policy components. Accordingly, recommendations for refining the policy system include optimizing the structure of policy instruments, enhancing the systemic integration of policies to improve institutional synergy, rationally planning policy timeframe, and expanding the coverage of policy targets.

## 1 Introduction

With the accelerating trend of global population aging, governments worldwide are facing mounting pressure on pension sustainability, healthcare systems, and long-term care (LTC) services. Within the framework of social long-term care insurance (LTCI) systems, pioneering countries such as Germany and Japan have established comprehensive LTCI systems. Through dedicated legislation, notably the Nursing Care Insurance Law, these countries have developed clearly define frameworks for financing, eligibility assessment, and service provision. Their experiences demonstrate that well-structured institutional arrangements can effectively achieve risk pooling and *ex ante* risk transfer (1, 2). In contrast, the United States has adopted a more market-oriented approach, combining private LTCI with public programs such as Medicaid to form a pluralistic and decentralized LTC system (3, 4). In recent years, reforms in countries such as South Korea and the Netherlands have further illustrated how diverse welfare state models integrate public financing, community-based care, and multi-level governance within LTC systems (5). Collectively, the successful experiences of these developed countries provide important insights and valuable lessons for the development of China’s LTC policy framework.

In China, the rapid expansion of the older adult population has intensified the urgency of establishing a robust LTCI system. By the end of 2023, China’s population aged 65 and older had reached 217 million, accounting for 15.4% of the total population. Moreover, the number of older adults with disabilities is projected to exceed 77 million by 2030 (6, 7). Since the launch of the initial pilot in Qingdao, Shandong Province, in 2012, China’s LTCI program has progressed from limited experimentation to a substantially expanded pilot phase. In 2020, the National Healthcare Security Administration and the Ministry of Finance issued the Guidance on Expanding the Pilot Program for the LTCI System. This policy document marked the formal transition of China’s LTCI initiative into a stage of accelerated nationwide promotion, and this phase is characterized by policy expansion and an exponential growth in related policy documents (8, 9).

In parallel with these institutional developments, academic research has gradually shifted from macro-level system design toward systematic evaluation of policy content and implementation outcomes. Existing studies primarily focus on analyses of early pilot policies (10–12), synthesizing experiences from pilot regions (13, 14), and comparative evaluations of different pilot models (15–18). However, systematic evaluations of LTCI policies themselves, particularly through policy scoring approaches, remain limited.

In recent years, policy text analysis based on policy instrument theory has been increasingly applied across a wide range of public policy studies. Policy instruments are defined as the tools and mechanisms employed by governments to achieve policy objectives and directly shape implementation effectiveness (19). This approach typically involves construction of two- or multidimensional analytical frameworks. For example, Wang et al. employed a two-dimensional framework of “policy instrument–policy rating” to analyze the composition, strengths, and weaknesses of LTCI policy instruments (20). In the domain of policy evaluation, the Policy Modeling Consistency (PMC) index model is widely used due to its relative objectivity in assessing internal policy coherence and multidimensional performance. Representative applications include the evaluation by Wang et al. of 16 pilot policies, which proposed strategies to enhance equity and sustainability (21). Similarly, Peng et al. assessed the sustainability of China’s LTCI system (22), while Duan et al. conducted a quantitative analysis of the diffusion characteristics of LTCI policies (23). Although these studies provide valuable insights, they tend to focus on specific policy dimensions and rarely examine the internal coordination between policy instrument configurations and structural differences across policy levels.

We, therefore, aimed to establish a two-dimensional analytical “policy instrument–policy level” framework to conduct a qualitative analysis of LTCI policy texts from the promotion phase. Furthermore, we integrate the PMC index model for policy scoring. This combined approach will substantively enrich research in the field of LTCI policy design and provide robust, evidence-based support for relevant decision-making authorities.

## 2 Methods

### 2.1 Data source and sample selection

Policy documents were retrieved using the keywords “long-term care” from the Peking University Magic Weapon database, with subsequent cross-verification conducted on the official websites of the National Healthcare Security Administration and relevant provincial and municipal governments. Given the evolutionary characteristics of LTCI policy, the current phase was identified as the policy promotion stage (2020–present). Policy texts from this stage comprehensively reflect the latest developments and future direction of the system implementation, thus possessing substantial analytical value. Consequently, the search period was limited to documents issued between January 1, 2020, and April 1, 2025. The preliminary search yielded 205 policy documents.

**Figure 1:**
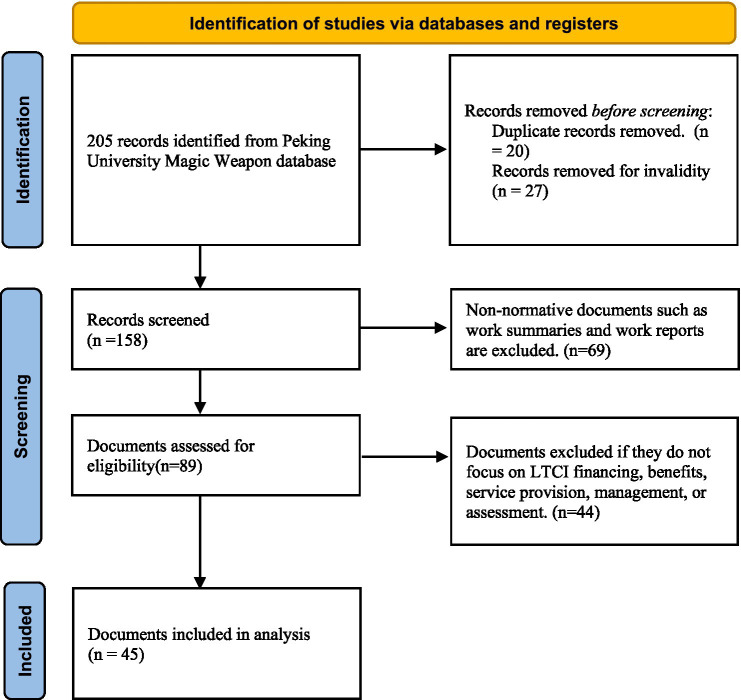
Data filtering procedure.

To ensure validity and relevance of the selected policy texts, the following rigorous inclusion criteria were established: (1) official documents issued by central or local government authorities and departments; (2) core content directly related to LTCI financing, benefits, service provision, management, or assessment; and (3) document types classified as normative instruments, such as implementation measures, guidance opinions, and pilot program plans. We excluded (1) non-normative documents, such as work summaries and reports; and (2) documents that were no longer in effect or constituted duplicate publications (Figure 1).

Following this rigorous screening process, a final sample of 45 documents meeting all established requirements was included (Supplementary materials). As detailed in Table 1, 11 documents were issued by national institutions (e.g., the National Healthcare Security Administration) and 34 by local governments, including provincial or municipal authorities.

**Table 1:** Summary of 45 policy texts included for analysis.

Policy level	Number of policies	Main issuing authorities	Core time period
Central	11 (24%)	National healthcare security administration, Ministry of finance, Ministry of Civil Affairs, etc.	2020–2024 (September 2024 was a policy-intensive period with five policies issued)
Local	35 (78%)	Healthcare security bureaus, Finance departments/bureaus, Health commissions of various provinces and cities, as well as their joint releases	2020–2024 (December 2020 and December 2021 were policy-intensive periods)
Total	45 (100%)	-	-

### 2.2 Two-dimensional analysis framework

**Figure 2:**
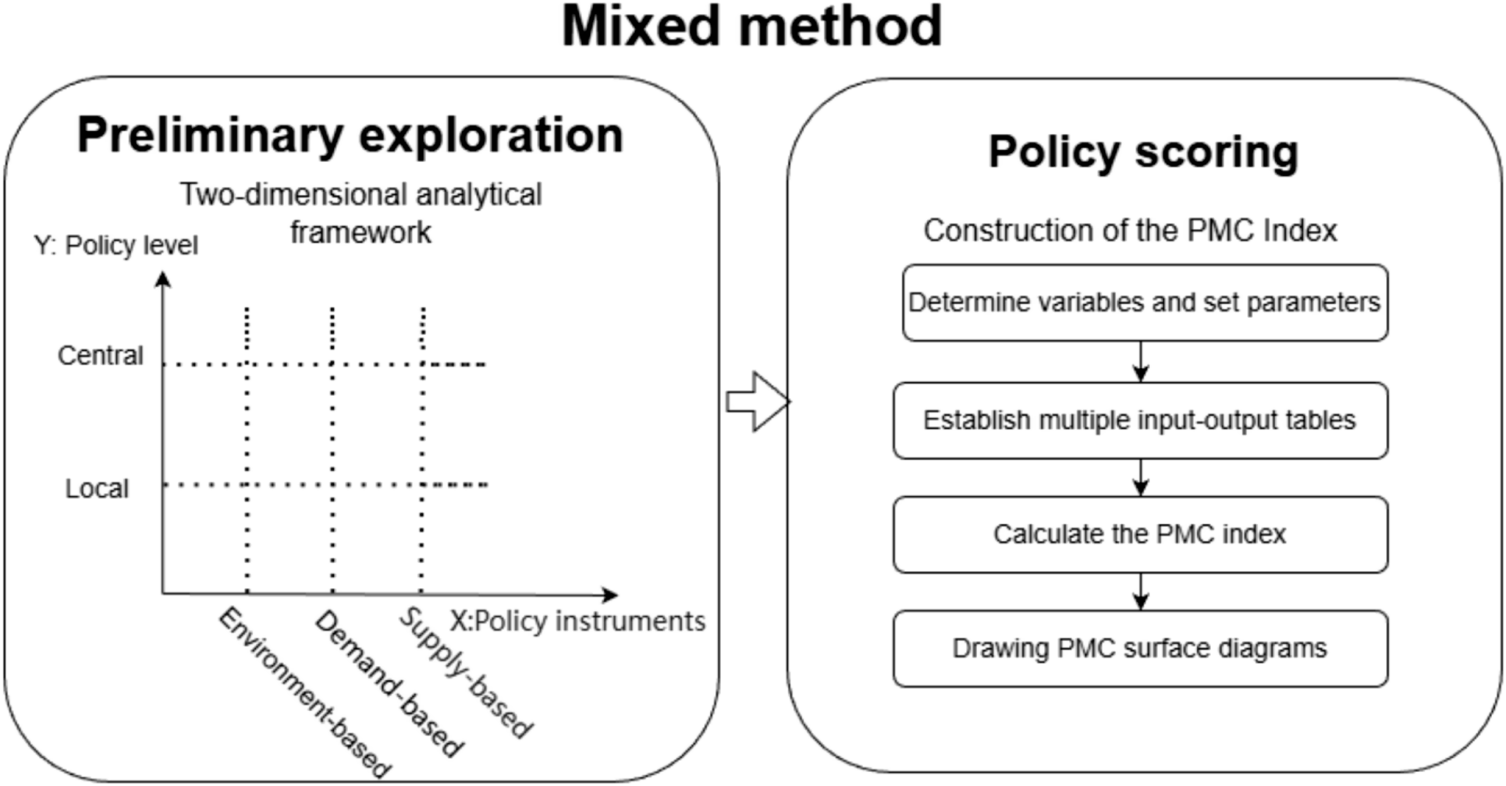
Research methods and design.

We established a two-dimensional analytical framework (Figure 2) with policy instruments as the X-dimension and policy level as the Y-dimension. Policies were categorized into central and local levels to investigate the differences in the application of various policy instruments between these two tiers. Central-level policies refer to those formulated by national authorities and administrative bodies for managing national affairs and safeguarding the overall interests of the country; local-level policies are enacted by provincial (autonomous regions, municipalities directly under the central government), municipal (prefecture-level cities), or other local governmental and administrative agencies to address specific affairs within their respective jurisdictions.

Policy instruments can be classified in various ways. Following the typology proposed by Rothwell and Zegveld, the instruments were categorized into supply-, demand-, and environment-based types, each of which was further subdivided into secondary instruments (24). Supply-based policy instruments focus on providing direct driving forces for the establishment of a LTC system, including talent supply, capital investment, digital construction, and infrastructure. Demand-based policy instruments aim to stimulate the market and drive the development of the LTC system through indirect means, such as promotion and encouragement, subsidies and funding, government purchasing, and public–private partnerships. Environment-based policy instruments seek to create a favorable policy environment to achieve the objectives of the LTC system, encompassing target planning, regulations and supervision, business coordination, and strategic measures. A detailed breakdown of the instruments is provided in Table 2.

**Table 2:** Coding basis for LTCI policies.

Instrument type	Secondary instrument and specific meaning
Supply-based	Talent supply, government supports talent team building to provide talent guarantee for LTCI. Capital investment, the government provides direct financial support to relevant parties in the long-term care system. Digital construction, the government provides technological support and digital infrastructure for the development of LTC. Infrastructure, the government establishes LTC service institutions and strengthens the construction of LTC facilities.
Demand-based	Promotion and encouragement, guide service providers to develop and promote various insurance products. Subsidies and funding, provide subsidies to all policy stakeholders to alleviate financial pressure. Government purchasing, promote market-oriented development through measures such as investing in third-party services. Public–private partnerships, government-enterprise collaboration to jointly provide LTC services, such as publicly-owned, privately-managed care institutions.
Environment-based	Target planning, a general description of the objectives and plans for the LTC system. Regulations and supervision, the government establishes relevant administrative regulations to standardize the behavior of all parties and promote industrial development. Business coordination, strengthen communication, coordination, and cooperation among all parties, and reinforce collaborative mechanisms. Strategic measures, various comprehensive measures implemented to further develop LTC.

Coding was independently performed by two researchers with a background in public health policy studies. Prior to formal coding, unified training and preliminary coding exercises were carried out to ensure a consistent understanding of the criteria. Discrepancies were addressed through group discussion until a consensus was reached. Using NVivo 20 software, a total of 370 coding nodes were generated. The consistency thereof was assessed using Cohen’s kappa coefficient, which yielded a value of 0.85, indicating a good level of inter-coder agreement.

### 2.3 PMC index model

The PMC index model, proposed by Ruiz-Estrada et al., is a policy evaluation tool grounded in the *omnia mobilis* (everything moves) assumption (25, 26). The model is employed to assess the internal consistency of individual policies, and visually presents the overall evaluation results and performance across dimensions via the PMC index and PMC surfaces (27). The PMC index model enables a comparatively objective analysis of a policy’s internal strengths and weaknesses across multiple dimensions, thereby mitigating subjectivity and ambiguity often inherent in traditional policy evaluations. It has been widely applied in policy assessments within fields such as healthcare and older adult care services (28, 29). The construction of the model in this study primarily involved three sequential steps.

First, variables were determined and parameters were set. Drawing upon the variable classifications proposed by Ruiz-Estrada and considering the specific content characteristics of the policies alongside existing scholarly research (19, 30–33), nine first-level variables were selected, including policy nature (X1), policy instrument (X2), policy timeframe (X3), policy level (X4), policy content (X5), incentives and constraints (X6), policy targets (X7), policy functions (X8) and policy supervision (X9). Each first-level variable encompasses several second-level variables. For instance, policy nature (X1) consists of 5 s-level variables: description (X1-1), advisory (X1-2), guidance (X1-3), support (X1-4), and prediction (X1-5). In total, 32 s-level variables were established (Table 3). Following a binary criterion, a value of 1 was assigned if the policy text content aligned with a second-level variable’s description; otherwise, a value of 0 was assigned. For example, if a policy text contained phrases such as “support social forces in establishing care institutions,” the second-level variable “X2-4 Infrastructure” was assigned a value of 1.

**Table 3:** Policy evaluation indicator system.

First-level variables	Second-level variables	Evaluation criteria	References
X1 Policy Nature	X1-1 Description	Determine whether the policy involves content related to description, recommendations, guidance, support, or forecasting.	Sun et al. (30)
	X1-2 Advisory		
	X1-3 Guidance		
	X1-4 Support		
	X1-5 Prediction		
X2 Policy Instrument	X2-1 Supply-Based	Determine whether the policy involves content related to supply-side, demand-side, or environmental tools.	Tang et al. (19)
	X2-2 Demand-Based		
	X2-3 Environment-Based		
X3 Policy Timeframe	X3-1 Short Term	Determine whether the policy’s timeframe involves short-term planning (1–2 years), medium-term planning (3–5 years), or long-term planning (over 5 years).	Xue et al. (31)
	X3-2 Medium Term		
	X3-3 Long Term		
X4 Policy Level	X4-1 Central Level	Determine whether the policy is issued at the central government level or implemented at the provincial/municipal level.	Policy content
	X4-2 Local Level		
X5 Policy Content	X5-1 Disability Assessment	Determine whether the policy content involves disability grading assessment, fund raising, procedural review, fund management, or benefit standards.	Policy content
	X5-2 Fund Raising		
	X5-3 Procedure Review		
	X5-4 Fund Management		
	X5-5 Benefit Standards		
X6 Incentives And Constraints	X6-1 Financial Subsidies	Determine whether the policy content involves fiscal subsidies, legal safeguards, talent cultivation, or risk management.	Zhang et al. (32)
	X6-2 Legal Protection		
	X6-3 Talent Supply		
	X6-4 Risk Management		
X7 Policy Targets	X7-1 Urban Workers	Determine whether the policy targets urban employees, rural residents, or no restrictions.	Xue et al. (31)
	X7-2 Rural And Urban Residents		
	X7-3 No Restrictions		
X8 Policy Functions	X8-1 Macro-Level Design	Determine whether the policy functions involve macro-level design, clarification of responsibilities and authorities, publicity and encouragement, or collaborative coordination.	Policy content
	X8-2 Clarification Of Responsibilities And Authorities		
	X8-3 Promotion And Encouragement		
	X8-4 Coordination And Collaboration		
X9 Policy Supervision	X9-1 Pre-Implementation Supervision	Determine whether the policy involves pre-implementation supervision, in-process supervision, or post-implementation supervision.	Chen et al. (33)
	X9-2 During-Implementation Supervision		
	X9-3 Post-Implementation Supervision		

Second, a multi-input–output table was constructed. Based on the correspondence between first-level and second-level variables, a multi-input–output table was developed (Table 4). This process effectively transformed qualitative policy content into a calculable data structure.

**Table 4:** Multiple input–output table.

First-level Variables	X1	X2	X3	X4	X5	X6	X7	X8	X9
Second-level Variables	X1-1	X2-1	X3-1	X4-1	X5-1	X6-1	X7-1	X8-1	X9-1
	X1-2				X5-2	X6-2		X8-2	
	X1-3	X2-2	X3-2	X4-2	X5-3	X6-3	X7-2	X8-3	X9-2
	X1-4	X2-3	X3-3		X5-4	X6-4		X8-4	
	X1-5				X5-5		X7-3		X9-3

The specific meanings of (X1-X9) are shown in Table 3.

Third, the PMC index was calculated, and PMC surfaces were plotted. The calculation of the PMC index proceeded in three stages: (1) Calculating second-level variable values, where Equations 1, 2 indicate that all second-level variables follow a [0, 1] distribution. (2) Calculating first-level variable values, where Equation 3 shows that the value of a first-level variable is the ratio of the sum of its second-level variable scores to the number of second-level variables. (3) Calculating the final PMC index value, where Equation 4 represents the sum of all first-level variable values (34). Furthermore, to facilitate comparison, the mean value of each first-level indicator was calculated. A “depression index” was also introduced to quantify deviations; its calculation is detailed in Equation 5.


X~N[0,1] (1)

X={XR:[0~1]} (2)

$$t(\sum \begin{array}{l} n\\ j=1 \end{array}\frac{X_{\bar{y}}}{T(X_{\bar{y}})}),t=1,2,3,4,5,6,7,8,9,10,\ldots$$ (3)

$$\mathrm{PMC}=[\begin{array}{ccc} X_{1}(\sum \begin{array}{l} 5\\ i=1 \end{array}\frac{X_{1i}}{5})+ & X_{2}(\sum \begin{array}{l} 3\\ j=1 \end{array}\frac{X_{2j}}{3})+ & X_{3}(\sum \begin{array}{l} 3\\ k=1 \end{array}\frac{X_{3k}}{3})+\\ X_{4}(\sum \begin{array}{l} 2\\ l=1 \end{array}\frac{X_{4l}}{2})+ & X_{5}(\sum \begin{array}{l} 5\\ m=1 \end{array}\frac{X_{5m}}{5})+ & X_{6}(\sum \begin{array}{l} 4\\ n=1 \end{array}\frac{X_{6n}}{4})+\\ X_{7}(\sum \begin{array}{l} 3\\ o=1 \end{array}\frac{X_{7o}}{3})+ & X_{8}(\sum \begin{array}{l} 4\\ p=1 \end{array}\frac{X_{8p}}{4})+ & X_{9}(\sum \begin{array}{l} 3\\ q=1 \end{array}\frac{X_{9q}}{3})\; \end{array}]$$ (4)

Depression Index=9−PMCIndex (5)

Based on the PMC index score, policies were classified into the following performance grades (35): Perfect (≥8), Excellent (7–8), Acceptable (5–7), and Poor (<5).

## 3 Results

### 3.1 Two-dimensional framework analysis

#### 3.1.1 Policy instrument dimension analysis

The distribution profile of policy instrument usage is presented in Table 5. Environment-based instruments were employed most frequently (181 counts, 48.9%). Within this category, Target planning (19.2%) and Regulations and Supervision (13.8%) constituted the primary tools, indicating a policy focus on establishing the institutional framework and clarifying authority and responsibility relationships. Among supply-based instruments, Digital Construction development (8.6%) and Talent Supply (7.0%) received considerable attention, which reflects the policy emphasis on IT infrastructure and professional workforce development. However, the relatively lower emphasis on capital investment (5.9%) may constrain the long-term sustainable provision of LTC services. Demand-based instruments were the least utilized overall (23.3%), with public–Private Partnerships accounting for only 1.9%. This finding suggests that the mobilization of market and social forces remains insufficient.

**Table 5:** Usage of LTCI policy instruments.

Instrument type	Secondary instrument	Frequency	Percentage	Total
Supply-based	Talent supply	26	7.0%	103 (27.8%)
	Capital investment	22	5.9%	
	Digital construction	32	8.6%	
	Infrastructure	23	6.2%	
Demand-based	Promotion and encouragement	31	8.4%	86 (23.3%)
	Subsidies and funding	31	8.4%	
	Government purchasing	17	4.6%	
	Public–private partnerships	7	1.9%	
Environment-based	Target planning	71	19.2%	181 (48.9%)
	Regulations and supervision	51	13.8%	
	Business coordination	23	6.2%	
	Strategic measures	36	9.7%	
Total	—	370	100%	370 (100%)

#### 3.1.2 Policy level dimension analysis

The distribution of policy instruments across central and local levels is detailed in Table 6. Statistically significant differences (*p* < 0.05) were observed between the central and local levels in their utilization of environment-based and demand-based policy instruments. Central-level policies demonstrated a comparatively balanced deployment of supply- (36.1%), demand- (29.2%), and environment-based (34.7%) instruments. Among these, Subsidies and Funding were the most frequently utilized (21.5%), reflecting the central government’s pivotal guiding role in establishing a nationwide institutional framework. In sharp contrast, local-level policies exhibited a pronounced reliance on environment-based instruments (51.0%), particularly Target Planning (21.0%) and Regulations and Supervision (13.2%). This pattern indicates that local governments primarily focus on refining implementation rules and adapting policies for localized deployment when executing central directives. Furthermore, local levels showed notably insufficient emphasis on instruments such as Infrastructure (2.3%) and public–Private Partnership (2.3%).

**Table 6:** Distribution of policy instruments by policy level.

Instrument type	Secondary instrument	Central level (*n*, %)	Local level (*n*, %)	Total (*n*, %)		*χ*^2^ test
				Central Level	Local Level	
Supply-based	Talent supply	12 (8.3%)	14 (5.4%)	52 (36.1%)	51 (19.8%)	*χ*^2^ = 11.96, *p* < 0.05
	Capital investment	5 (3.5%)	17 (6.6%)			
	Digital construction	18 (12.5%)	14 (5.4%)			
	Infrastructure	17 (11.5%)	6 (2.3%)			
Demand-based	Promotion and encouragement	9 (6.3%)	22 (8.6%)	42 (29.2%)	75 (29.2%)	*χ^2^* ≈ 0.0, *p* > 0.05
	Subsidies and funding	31 (21.5%)	30 (11.7%)			
	Government purchasing	1 (0.7%)	17 (6.6%)			
	Public–private partnerships	1 (0.7%)	6 (2.3%)			
Environment-based	Target planning	17 (11.8%)	54 (21.0%)	50 (34.7%)	131 (51.0%)	*χ*^2^ = 9.84, *p* < 0.05
	Regulations and supervision	17 (11.8%)	34 (13.2%)			
	Business coordination	6 (4.2%)	17 (6.6%)			
	Strategic measures	10 (6.9%)	26 (10.1%)			
Total	—	144 (100%)	257 (100%)	144 (100%)	257 (100%)	—

### 3.2 Policy scoring evaluation of LTCI policies

#### 3.2.1 Analysis of overall policy characteristics

As presented in Table 7, we quantified the scores of the 45 policies (P1–45) across the nine first-level variables (X1–9), calculating the corresponding PMC index and policy grade for each. The specific scoring details can be found in the Supplementary materials. Overall, China’s LTCI-related policies demonstrated an acceptable level of performance, although some policies performed poorly. The average PMC index for the 45 policy texts was 6.01, corresponding to the Acceptable grade. Of these, 28 policies (62%) were graded as Acceptable (5 ≤ PMC index < 7), eight policies (18%) as Excellent (7 ≤ PMC index < 8), and nine policies (20%) as Poor (< 5). Policy P13 achieved the highest PMC index (7.83), corresponding to the Excellent grade, while P23 received the lowest (3.30), corresponding to the Poor grade. This distribution of policy scores reflects significant heterogeneity in the completeness of policy design across different regions and administrative levels.

**Table 7:** Scores of 45 LTCI policies.

Policy code	X1	X2	X3	X4	X5	X6	X7	X8	X9	PMC index	Depression index	Policy grade	Policy level
P1	0.80	0.67	0.67	0.50	1.00	0.50	0.67	1.00	1.00	6.80	2.20	Acceptable	Local
P2	0.40	1.00	0.33	0.50	1.00	0.50	0.33	0.50	1.00	5.57	3.43	Acceptable	Local
P3	0.40	1.00	0.33	0.50	0.40	0.50	0.00	0.67	1.00	4.80	4.20	Poor	Local
…													
P22	0.80	0.67	0.33	0.50	0.40	0.50	0.33	0.75	0.00	4.28	4.72	Poor	Local
P23	0.80	0.33	0.33	0.50	1.00	0.00	0.33	0.00	0.00	3.30	5.70	Poor	Local
P24	0.80	1.00	0.00	0.50	1.00	1.00	0.67	1.00	1.00	6.97	2.03	Acceptable	Local
…													
P44	1.00	0.67	0.33	0.50	0.20	0.50	0.33	1.00	0.33	4.86	4.14	Poor	Central
P45	0.80	0.67	0.33	0.50	0.60	0.50	0.33	0.75	0.67	5.15	3.85	Acceptable	Central
Mean ± Std	0.78 ± 0.19	0.89 ± 0.17	0.33 ± 0.19	0.5 ± 0	0.77 ± 0.28	0.69 ± 0.27	0.39 ± 0.22	0.82 ± 0.22	0.83 ± 0.3	6.01 ± 1.1	2.99 ± 1.1	Acceptable	—

#### 3.2.2 First-level variables analysis

**Figure 3:**
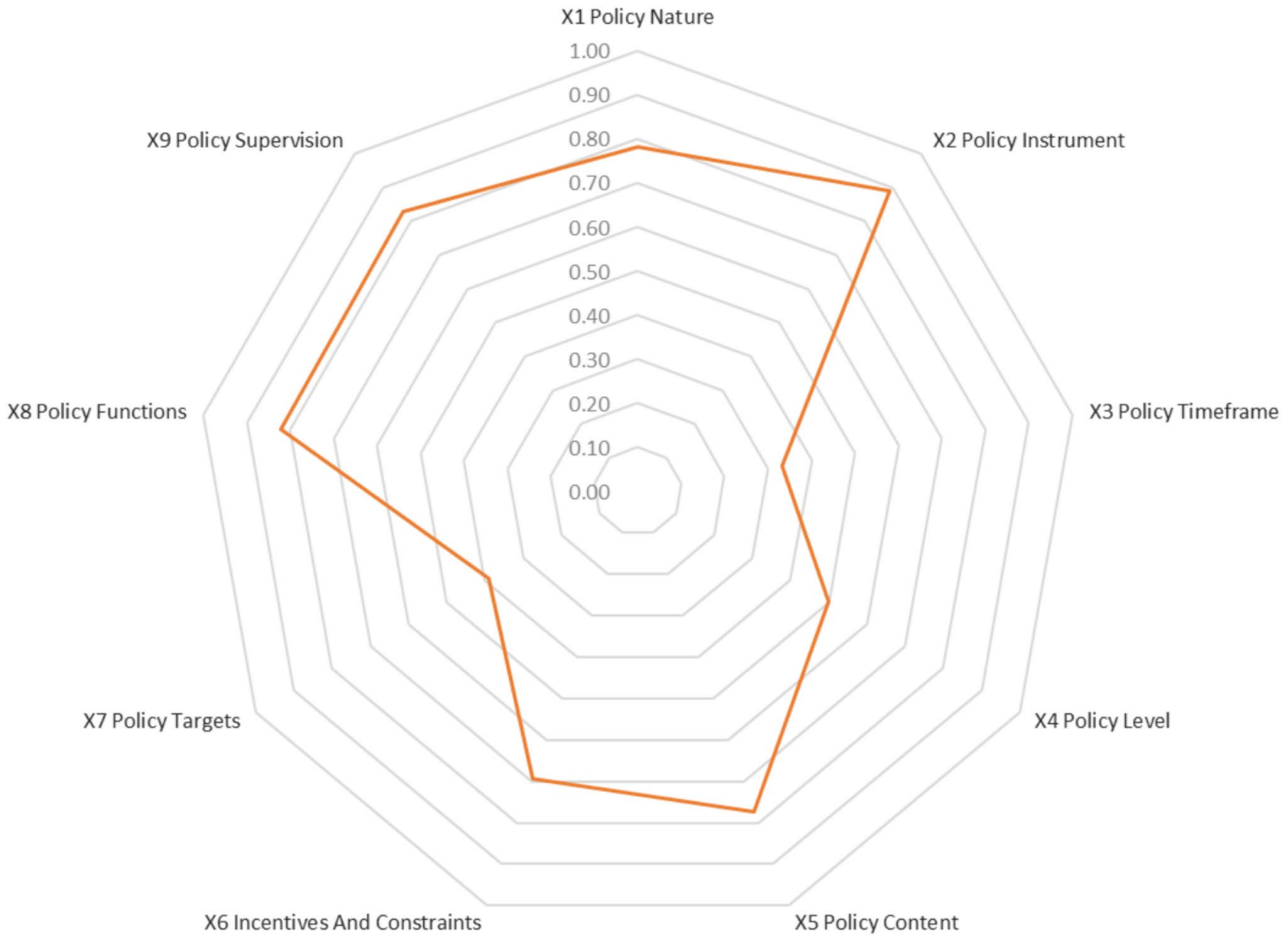
Radar chart of the average scores of the 45 policies on the first-level variables.

A radar chart depicting the average scores of the 45 policies across the first-level variables (Figure 3) revealed conspicuously weak performance in X7 (Policy Targets, mean 0.39), X3 (Policy Timeframe, mean 0.33), and X4 (Policy Level, mean 0.50). The coverage of policy targets (X7) is limited, primarily focusing on urban employees (approximately 51%), with insufficient coverage for urban and rural residents (approximately 28%) and other groups. Policy timeframe (X3) is predominantly short- to medium-term (1–5 years), which results in a lack of long-term institutional planning. The dominance of local-level policies indicates an imperative need to strengthen the overall planning and guiding role of central-level policies. In sharp contrast, policies performed commendably well in X2 (Policy Instruments), X8 (Policy Functions), and X9 (Policy Supervision), with mean scores all exceeding 0.80. This indicates that current policies are sophisticated in terms of instrument diversity, functional clarity, and supervision mechanism design.

#### 3.2.3 Representative policies analysis

**Figure 4:**
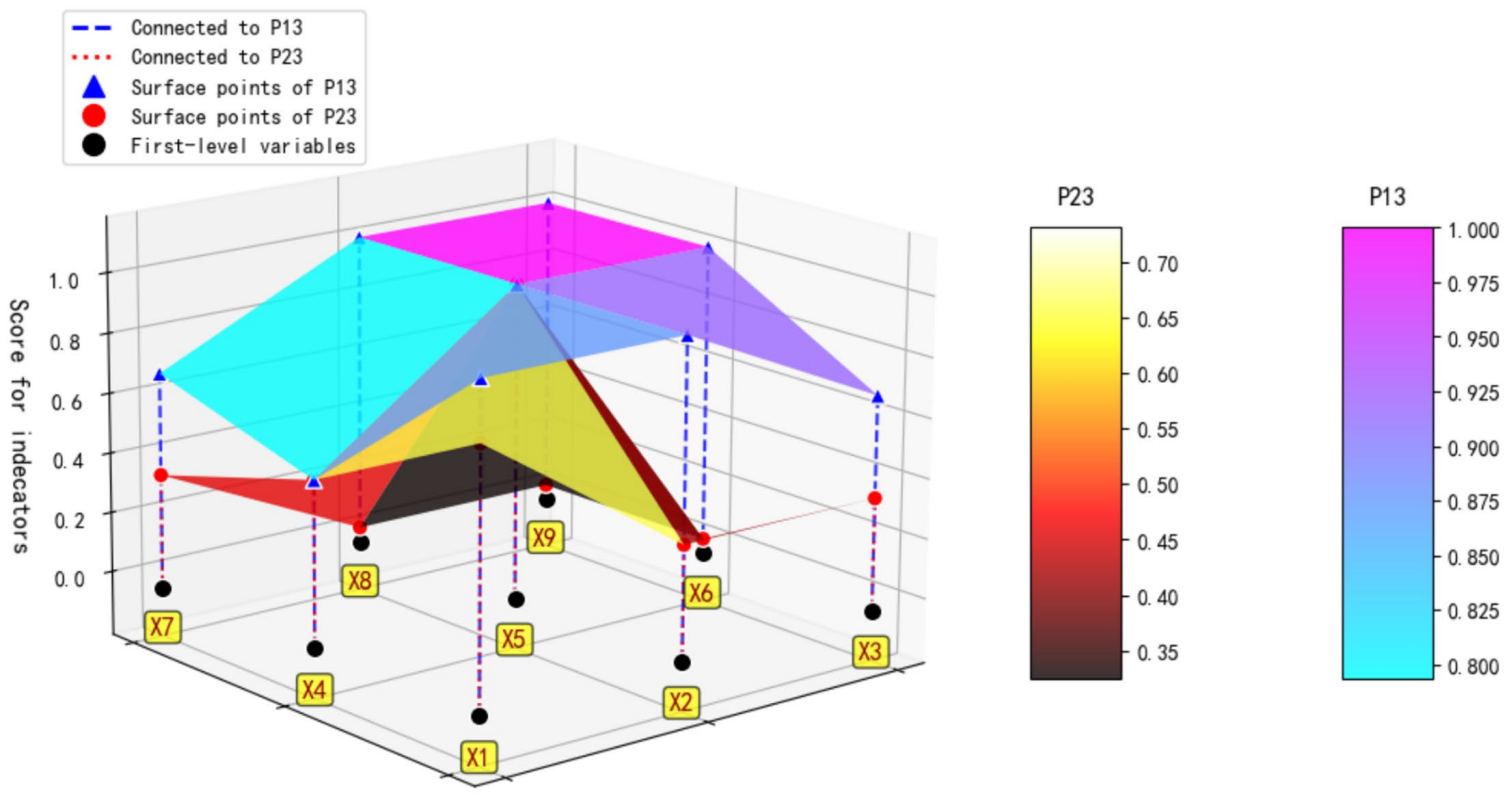
PMC surface chart of P13 and P23.

To elucidate the sources of policy variation, a comparative analysis was conducted on P13 (Zhoushan City), which exhibited the highest PMC index, and P23 (Nanning City), which received the lowest. Their PMC surfaces are plotted in Figure 4. Additionally, Policy P17 (Fuzhou City), with a medium score, was selected for supplementary analysis to reflect the design characteristics typical of policies in this range.

P13 scored highly on all variables except X3 (Policy Timeframe) and X7 (Policy Targets). Its policy design is characterized by systematic, pluralistic, and coordinated features. As a region with a developed economy and a mature insurance market, Zhoushan possesses the institutional foundation for resource integration and innovation, enabling proactive exploration in areas such as multi-level security, diversified financing, and comprehensive supervision.

In sharp contrast, P23 scored exceptionally low on X2 (Policy Instruments), X6 (Incentives and Constraints), X8 (Policy Function), and X9 (Policy Supervision), indicating fundamental shortcomings in its policy design. The Nanning pilot program is still in an exploratory stage, with the policy focus limited to promoting enrollment among specific unit-based groups. Systematic development in instrument application, incentive mechanisms, inter-departmental collaboration, and supervision systems have not yet been fully implemented, reflecting a practical logic of “launch first, refine later” under conditions of limited resources in the pilot area.

The policy differences between the two locations profoundly underscore the variations in regional economic development levels, institutional development stages, and policy targeting. Zhoushan represents a “systematic construction” model, while the Nanning pilot exemplifies an “incremental exploration” pathway. This provides robust empirical evidence for the categorized advancement and differentiated guidance of China’s LTCI system.

Policy P17, with a PMC index of 6.18 (ranking 23rd), shows similar deficiencies in X3 (Policy Timeframe) and X7 (Policy Targets) but performs well in other aspects, demonstrating acceptable internal consistency in its design. As a second-batch pilot city, Fuzhou’s policy targets differ significantly from the previous two cases, primarily covering employees enrolled in basic medical insurance. It adopts a prudent strategy of first covering the “core group” and then gradually expanding coverage.

## 4 Discussion

We systematically evaluated China’s LTCI policies through a two-dimensional framework and the PMC index model. Structural imbalance exists in the use of policy instruments, characterized by over-reliance on environment-based instruments and a relative insufficiency of supply-based and demand-based tools. This imbalance is pronounced in areas such as capital investment and public–private partnerships.

As crucial levers for governments to achieve governance objectives, the rational configuration of policy instruments directly impacts the effectiveness and sustainability of policy implementation (36). Our dimensional analysis reveals that the excessive proportion of environment-based instruments, coupled with the underutilization of supply-based and demand-based tools, not only constrains the comprehensive achievement of policy goals but may also trigger a series of derivative issues in long-term systemic development. This finding is consistent with prior research (19, 37).

It is crucial to clarify that the “over-reliance” on environment-based instruments does not negate their intrinsic importance, but rather indicates their disproportionately dominant position within the policy instrument mix. This is manifested as the construction of an institutional framework through indirect means such as target planning, regulations, and supervision, which compresses flexible space for applying supply-based and demand-based instruments. Consequently, the capacity of supply-based instruments—which provide direct “hardware” and “software” support such as funding, technology, talent, and public services to establish the material foundation for policy goals—is significantly weakened. Simultaneously, demand-based instruments have failed to fully activate or mobilize the vitality of social resource allocation, leading to an ineffective linkage between policy objectives and market mechanisms.

The causes of this structural imbalance in policy instruments can be attributed to two main factors. First, the imbalance is partly rooted in the “incrementalism” that characterizes China’s policymaking process. The adoption of incrementalism as a primary decision-making model facilitates the formulation of more feasible policies and helps minimize implementation errors (38). The progression of LTCI from regional pilots to expanded trials and nationwide implementation represents a complex and enormous undertaking. This process involves the construction and coordination of multiple dimensions (including the policy system, fiscal budgets, and infrastructure) and entails the interest negotiation among decision-makers representing different stakeholder groups. A series of incremental reforms allows these decision-makers to continuously achieve consensus, thereby facilitating the smooth formulation and adoption of policies. Second, the imbalance also stems from the “rationalist” behavioral strategies of local governments. Local governments rationally conduct cost-effectiveness analyses to determine optimal solutions. Crucially, however, such decision-making often prioritizes maximizing benefits within their own jurisdictions. From a national perspective, this localized rationality can aggregate into a risk of structural imbalance across the broader policy landscape.

Our results further indicate that central and local governments exhibit distinct priorities in their use of policy instruments. Central policies demonstrate greater balance and guidance, whereas local policies tend to prioritize environmental shaping and rule specification. However, the systemic coordination between the two levels urgently requires strengthening. As the top-level designer of the LTCI policy, the central government’s core objectives are to establish a nationally unified institutional framework and ensure the scientific rigor and equity of the policy direction. Consequently, its selection of policy instruments emphasizes balance and guidance (39). Specifically, the central level formulates nationwide pilot guidance to clarify the fundamental principles for core elements such as coverage, financing standards, and benefit levels of LTCI. This aims to preclude regional policy deviations that could lead to either excessive or insufficient provision.

Furthermore, through the deployment of instruments such as fiscal subsidies and pilot demonstrations, the central government incentivizes local policy practice and steers local initiatives toward alignment with the national framework, thereby safeguarding the systemic integrity of the LTCI program (40). In contrast, local governments, as the primary implementers of LTCI policies, must tailor policies to local conditions, including socio-economic development levels, demographic structures, and the distribution of care resources. Consequently, they lean more heavily on environment-based instruments for environmental shaping and rule specification. For instance, localities enact regional regulations and management measures to set accreditation standards for care institutions and specify qualification requirements for care personnel, thereby fostering an orderly market environment for LTCI implementation. They also determine fee schedules for care services and delineate service areas based on local factors such as the distribution of disabled older adult and cost-of-living indices.

Nevertheless, significant shortcomings persist in the coordination between central and local LTCI policies. Local governments may misinterpret the core intent of central directives during the rule-specification process, leading to policy deviations from central requirements. Conversely, the mechanisms for information sharing and collaboration between levels are underdeveloped. The central government often lacks real-time insights into the challenges and experiences encountered during local implementation. Simultaneously, local governments may not receive timely central guidance when making policy adjustments. This disconnect hinders the rapid translation of successful pilot innovations into nationwide institutional practices. Moreover, it contributes to policy fragmentation in some regions, which is detrimental to the unified national advancement of the LTCI system (41, 42).

The PMC index evaluation indicates that while China’s LTCI policies demonstrate an acceptable overall performance, significant room for improvement remains in areas such as policy target coverage, policy timeframe planning, and inter-level policy coordination. As an important diagnostic tool utilized to gage policy quality, the PMC index model provides a multidimensional assessment of LTCI policies during their promotion phase. The results, showing an overall acceptable performance, are attributable to scientifically sound designs in core areas such as the financing mechanism, service provision framework, and pilot implementation models, which have effectively responded to the LTC security needs of the disabled population amid population aging (43). However, the current LTCI policy target coverage focuses on older adults with disabilities. Moreover, most pilot regions tie eligibility for LTCI to enrollment in either the employee basic medical insurance or the urban and rural resident basic medical insurance, thereby systemically excluding certain population groups. The care needs of mildly or moderately disabled older adults are not yet fully addressed. This group often faces a dilemma between “inadequate care services” and “not meeting the severe disability assessment criteria,” resulting in a coverage gap within the policy safety net (44, 45). In contrast, specific populations not enrolled in basic medical insurance—such as flexible workers, new-economy practitioners, and left-behind older adults in rural areas—find it challenging to be included in the LTCI scheme, indicating an imperative need to enhance the policy’s inclusivity.

Furthermore, LTCI policy remains in a pilot exploration stage. Most regional policy documents are issued as “pilot implementation plans,” which are often characterized by short-term validity cycles and a lack of clear long-term planning. This leads to uncertainty regarding policy continuity once pilot periods expire, revealing a tendency toward short-sightedness in long-term institutional design. Failure to fully account for long-term variables such as the pace of population aging and rising care costs, coupled with the absence of a dynamic optimization plan for adjusting financing standards and benefit levels, may result in future issues such as imbalances in fund revenues and expenditures and a disconnect between protection levels and actual needs.

Our comparative analysis of representative policies reveals that regional economic development stages, institutional foundations, and policy targeting are significant determinants influencing policy design. First, the stage of economic development serves as the material foundation for LTCI policy implementation. It directly determines a local government’s fiscal capacity to invest in the security system, the willingness of market entities to participate, and the affordability of contributions for residents, thereby influencing the breadth and depth of policy design. Zhoushan City, with its developed economy and mature insurance market, possesses substantial fiscal and healthcare fund reserves. This enables support for a multi-tiered system covering both employees and urban/rural residents across the entire region, achieving diversified financing from fiscal budgets, healthcare funds, employers, individuals, and commercial insurance, with correspondingly more varied service models. Fuzhou City, while economically robust, does not match Zhoushan’s level of maturity. Its policy relies primarily on the employee medical insurance system for financing, prioritizing the care needs of disabled employees, thus resulting in more constrained financing channels and coverage. The pilot area in Nanning operates under constrained economic conditions with limited surplus in healthcare funds and fiscal budgets. To mitigate financial risks, the pilot program is restricted to a narrow scope covering specific central and regional institutions stationed in Nanning. It does not establish a unified financing mechanism and only provides basic care benefits.

Second, LTCI does not exist as an independent system; its design must build upon existing local institutional foundations, including the medical insurance administration system, care service networks, and disability assessment mechanisms. The robustness of these foundations directly determines the starting point and implementation pathway of the policy. Zhoushan City has already established a comprehensive urban and rural medical insurance administration network and a diversified care service system. This solid institutional base allows for direct implementation of full regional coverage and the immediate establishment of a multi-level protection model. Fuzhou City, as a national pilot, has developed administration and assessment systems tailored for the employee population but currently lacks the capacity for universal service provision. Consequently, it has adopted a gradualist path, first covering employees with disabilities before expanding to those with moderate disabilities. The pilot in Nanning has yet to establish mature care service and assessment systems and lacks experience in cross-population medical insurance coordination. It is therefore limited to small-scale experimentation within specific units to validate basic administrative processes, resulting in a conservative and narrowly scoped initial policy phase.

Finally, distinct policy targeting directs local governments to prioritize different population groups, care priorities, and institutional functions in their policy design, resulting in the formation of differentiated policy frameworks. Zhoushan’s policy goal is to build a universal, multi-level LTC security system, with its core orientation being inclusiveness and diversification. Accordingly, its policy design covers all basic medical insurance participants, preserves the fundamental coverage of policy-oriented LTCI, and introduces commercial long-term care insurance to address personalized demands. Fuzhou’s policy goal is to fulfill the national pilot mandate and provide replicable operational experience for employee LTCI nationwide, with its core orientation being standardization and demonstration. Thus, its policy design prioritizes the employee group, which has a mature contribution mechanism and well-established information management systems, first addressing the core needs of the severely disabled before gradually expanding coverage. The policy goal of the Nanning pilot in Guangxi is to verify the implementation feasibility of LTCI policy within a limited scope, with its core orientation being “low-risk” and “exploratory.” Consequently, its policy covers only the specific group of central and regional units stationed in Nanning, focusing on minimizing pilot risks and accumulating basic operational data, which renders its design distinctly exploratory and conservative.

### 4.1 Future directions

Based on the above conclusions, several policy optimization recommendations are proposed. First, the structure of policy instruments should be optimized to facilitate a more balanced policy instrument mix. The proportion of environment-based instruments should be appropriately moderated, while the synergistic deployment of demand-based and supply-based instruments should be strengthened to mitigate potential inefficiencies associated with over-reliance on a single category of policy instrument. Market demand can be stimulated by expanding government procurement of services, promoting public–private partnership models, and encouraging greater participation of commercial insurance. Supply capacity can be enhanced by establishing dedicated funding mechanisms for care personnel training, improving service facilities in remote areas, and enhancing the precision of capital investment. Existing research indicates that, despite overall improvements in LTC resource allocation in China, substantial regional disparities remain, with an estimated shortage of approximately 330,000 beds and 1.61 million care workers (46). Concurrently, the national pilot program for vocational skill certification for “Long-Term Care Specialists” has been launched. These specialists are expected to constitute a critical component of the workforce, providing essential human capital support and helping to ensure service quality in the implementation of the LTCI system. During the policy promotion phase, the use of supply-based policy instruments is likely to increase, and the overall policy instrument structure is expected to gradually approach a more stable and balanced configuration as the system continues to mature.

Second, the systemic integration of policies should be strengthened to enhance institutional constructive collaboration. The central government should further reinforce top-level institutional design by formulating unified standards for disability assessment, service specifications, and guidelines for information-platform development. At the local level, policies should not only implement central directives but also adapt them in innovative ways based on local conditions to prevent policy fragmentation. Establishing a policy system characterized by “central coordination, local innovation, and vertical linkage” will significantly enhance the overall effectiveness of the LTCI system. In 2021, the National Healthcare Security Administration, in collaboration with the Ministry of Civil Affairs, issued the *Notice on the Standards for Disability Assessment for Long-Term Care (Trial)*, marking the release of the first nationwide unified standard for long-term care disability assessment and representing a critical milestone in the development of China’s LTCI system (47). Furthermore, with ongoing advancements in information technology and the refinement of LTCI-related policies, intelligent and mobile management systems tailored to LTCI operations have begun to emerge in the market. These systems support LTCI service institutions, personnel, and government regulatory authorities, thereby facilitating information sharing and enhancing coordination between central and local governments.

Third, the design of policy timeframe should be improved to strengthen institutional sustainability. While consolidating short- and medium-term policies, efforts should be progressively intensified to develop long-term institutional planning that clarifies policy objectives, implementation pathways, and stage-specific evaluation mechanisms. Data indicate that, in 2024, the number of participants in the LTCI pilot reached 187.8634 million across 49 national pilot cities, with 1.4625 million beneficiaries. The LTCI fund generated revenues of 27.96 billion yuan, while expenditures amounted to 13.108 billion yuan. There were 8,837 designated LTCI service institutions and 292,800 care service personnel (48). Compared with the initial pilot phase, the institutional foundation has gradually been consolidated, thereby objectively promoting the robust development of economy. The development of the LTCI system has now entered an accelerated phase characterized by a focus on quality improvement and efficiency enhancement. Therefore, in pilot regions, a three-stage promotion strategy of “pilot–expansion–deepening” can be adopted to facilitate a smooth transition from localized experimentation to comprehensive system development.

Fourth, the coverage of policy targets should be expanded to advance institutional equity. In regions with higher levels of economic development and more mature system operations, active efforts should be made to include urban and rural residents, as well as flexible workers, within the coverage scope, thereby gradually advancing toward universal coverage. Currently, Zhejiang Province and Shanghai have achieved full-population coverage for both employees and urban and rural residents, improving the quality of life of disabled individuals and reducing the financial burden on their families. For regions in the early stages of pilot implementation, the insured population can be expanded gradually and in a risk-controlled manner to enhance the inclusiveness and social equity of the system.

### 4.2 Limitations

Despite providing a systematic evaluation of China’s LTCI policies, this study has some limitations. First, our study constitutes a static cross-sectional analysis, which limits its capacity to capture the dynamic evolution of policies. The exclusion of policies published before 2020 may affect the composition of the final sample and introduce bias into interpretations of policy evolution. Furthermore, potential bias within the selected sample of 45 policy documents may compromise the representativeness of the evaluation, for example by inflating overall policy scores. Second, because this study focuses exclusively on policy texts, it cannot fully uncover potential gaps between policy design and actual implementation. The refinement of policy texts does not necessarily translate into effective implementation`, a factor that should be considered when interpreting the scoring results. Policy texts often present an idealized institutional framework but may overlook practical constraints in local implementation, such as resource disparities or inter-departmental coordination barriers. For instance, in some LTCI pilot regions, fiscal constraints have resulted in reduced service coverage, thereby creating a significant gap between actual practice and the universal goals articulated in policy documents. Third, although efforts were made to ensure objectivity in the scoring process, a certain degree of subjective judgment was unavoidable, particularly when assessing qualitative content. Finally, the PMC index model applied in this study adopts an equal-weight design, which fails to capture the actual differences in importance among policy variables, thereby weakening the discriminative power of the evaluation results. Additionally, the binary coding scheme (0 or 1) may oversimplify policy complexity, as it cannot capture gradations of policy intensity or comprehensiveness. Future research could address these limitations by incorporating a broader sample and extending the analysis to dynamic, longitudinal designs that track policy evolution. Methods such as the Delphi technique could be applied to assign differentiated weights to model variables, thereby developing an evaluation system that better reflects the distinctive characteristics of the policies under study.

## Data Availability

The original contributions presented in the study are included in the article/Supplementary material, further inquiries can be directed to the corresponding author.
