# Supplementary material for: Evaluating long-term care insurance policies in China: an integrated analysis of policy instruments and PMC index model
Source: Front Public Health. 2026 Jan 13;13:1740854. doi: 10.3389/fpubh.2025.1740854 (PMC12834800; doi:10.3389/fpubh.2025.1740854)
Supplement: Supplementary file 1 [file Table_1.DOCX]

List of Tables

[**Table1 The 45 policy texts included for analysis.** 2](#_Toc215923771)

[**Table2 The scores of 45 policy texts** 5](#_Toc215923772)

**Table1 The 45 policy texts included for analysis.**

| **Policy code** | **Policy Document Name** | **Issuing Authority** | **Date** | **Policy level** |
| --- | --- | --- | --- | --- |
| P1 | Notice on Issuing the "Beijing Municipal Long-Term Care Insurance System Pilot Expansion Plan" | Beijing Municipal Medical Insurance Bureau, etc. | 2020/10/28 | Local (Provincial) |
| P2 | Notice on Issuing the "Detailed Implementation Rules for the Tianjin Municipal Long-Term Care Insurance System Pilot Implementation Plan (Trial)" | Tianjin Municipal Medical Insurance Bureau, etc. | 2021/01/22 | Local (Provincial) |
| P3 | Notice on Issuing the "Management Measures for Designated Long-Term Care Insurance Nursing Institutions in Tianjin" | Tianjin Municipal Medical Insurance Bureau | 2023/05/04 | Local (Provincial) |
| P4 | Notice on Issuing the "Hohhot City Long-Term Care Insurance System Pilot Implementation Plan" | Hohhot Municipal People's Government Office | 2020/12/31 | Local (Municipal) |
| P5 | Notice on Issuing the Implementation Plan for Panjin City's Participation in the National Long-Term Care Insurance System Pilot Work | Panjin Municipal People's Government Office | 2020/12/15 | Local (Municipal) |
| P6 | Notice on Issuing the "Jilin Province Implementation Plan for Deepening the Long-Term Care Insurance System Pilot Work" | Jilin Provincial Medical Insurance Bureau | 2021/04/13 | Local (Provincial) |
| P7 | Notice on Issuing the "Guiding Opinions on Conducting Pilot Work for Long-Term Care Insurance Home Care Services" | Jilin Provincial Medical Insurance Bureau | 2022/11/8 | Local (Provincial) |
| P8 | Notice on Issuing the "Implementation Opinions on Further Standardizing the Long-Term Care Insurance Pilot Work in this District (Trial)" | Shanghai Jiading District People's Government | 2022/03/23 | Local (District) |
| P9 | Notice on Issuing the "Pudong New Area Long-Term Care Insurance Supervision and Management Measures (Trial)" | Shanghai Pudong New Area People's Government | 2020/11/08 | Local (District) |
| P10 | Notice of the Suzhou Municipal Government on Issuing the Implementation Opinions for the Second Phase of the Long-Term Care Insurance Pilot Work | Suzhou Municipal People's Government | 2020/01/17 | Local (Municipal) |
| P11 | Notice on Issuing the "Xuzhou City Long-Term Care Insurance Implementation Rules" | Xuzhou Municipal Medical Insurance Bureau | 2021/11/12 | Local (Municipal) |
| P12 | Notice of the Zhejiang Provincial Medical Insurance Bureau and Zhejiang Provincial Finance Department on Issuing the "Guiding Opinions on Deepening the Long-Term Care Insurance System Pilot" | Zhejiang Provincial Medical Insurance Bureau | 2022/06/02 | Local (Provincial) |
| P13 | Notice on Issuing the "Zhoushan City Implementation Plan for Building a Multi-level Long-Term Care Security System" | Zhoushan Municipal Government Office | 2024/03/08 | Local (Municipal) |
| P14 | Notice on Issuing the "Hangzhou City Long-Term Care Insurance Trial Measures" | Hangzhou Municipal People's Government Office | 2024/10/15 | Local (Municipal) |
| P15 | Issuance of the Implementation Opinions on the "Anqing City Urban Employee Long-Term Care Insurance Pilot" | Anqing Municipal People's Government Office | 2020/01/05 | Local (Municipal) |
| P16 | Notice of the Fuzhou Municipal People's Government on Issuing the Implementation Plan for the Long-Term Care Insurance System Pilot | Fuzhou Municipal People's Government | 2020/12/21 | Local (Municipal) |
| P17 | Notice on Issuing the "Fuzhou City Long-Term Care Insurance Implementation Rules" | Fuzhou Municipal Medical Insurance Bureau, etc. | 2021/03/26 | Local (Municipal) |
| P18 | Notice on Revising and Issuing the "Kaifeng City Long-Term Care Insurance System Trial Measures" | Kaifeng Municipal People's Government | 2021/07/30 | Local (Municipal) |
| P19 | Notice on Issuing the "Xiangtan City Long-Term Care Insurance System Pilot Implementation Plan" | Xiangtan Municipal People's Government | 2020/12/10 | Local (Municipal) |
| P20 | Notice on Issuing the "Guangzhou City Long-Term Care Insurance Trial Measures" | Guangzhou Municipal Medical Insurance Bureau, etc. | 2024/01/03 | Local (Municipal) |
| P21 | Notice on Issuing the Guangzhou City Long-Term Care Insurance Service Management Regulations | Guangzhou Municipal Medical Insurance Bureau, etc. | 2023/12/08 | Local (Municipal) |
| P22 | Notice on Central and District-Direct Units Stationed in Nanning Participating in the Nanning City Long-Term Care Insurance System Pilot | Guangxi Zhuang Autonomous Region Medical Insurance Bureau | 2021/12/07 | Local (Provincial) |
| P23 | Notice on Further Promoting the Participation of Central and District-Direct Units Stationed in Nanning in the Nanning City Long-Term Care Insurance System Pilot | Guangxi Zhuang Autonomous Region Medical Insurance Service Center | 2024/03/25 | Local (Institutional Unit) |
| P24 | Notice on Issuing the "Chengdu City Long-Term Care Insurance Implementation Rules" | Chengdu Municipal Medical Insurance Bureau, etc. | 2022/06/01 | Local (Municipal) |
| P25 | Implementation Opinions of the Chengdu Municipal People's Government on Carrying out a New Round of Long-Term Care Insurance Reform | Chengdu Municipal People's Government | 2022/06/07 | Local (Municipal) |
| P26 | Notice on Issuing the "Qianxinan Prefecture Long-Term Care Insurance Trial Implementation Rules" | Qianxinan Prefecture Medical Insurance Bureau | 2020/12/28 | Local (Municipal) |
| P27 | Notice of the Kunming Municipal People's Government on Issuing the Comprehensive Pilot Work Plan for the Long-Term Care Insurance System (Revised Version) | Kunming Municipal People's Government | 2023/02/14 | Local (Municipal) |
| P28 | Notice on Issuing the "Management Measures for Designated Service Institutions under Kunming City's Long-Term Care Insurance (Trial)" | Kunming Municipal Medical Insurance Bureau | 2024/03/18 | Local (Municipal) |
| P29 | Notice on Issuing the Hanzhong City Long-Term Care Insurance Implementation Measures (Trial) | Hanzhong Municipal People's Government Office | 2020/11/23 | Local (Municipal) |
| P30 | Notice on Issuing the "Urumqi City Long-Term Care Insurance Measures" | Urumqi Municipal People's Government Office | 2021/12/15 | Local (Municipal) |
| P31 | Implementation Opinions of the Chongqing Medical Insurance Bureau and Chongqing Finance Bureau on Expanding the Long-Term Care Insurance System Pilot | Chongqing Municipal Medical Insurance Bureau, etc. | 2021/11/22 | Local (Provincial) |
| P32 | Notice on Issuing the "Heze City Employee Long-Term Care Insurance Implementation Measures" | Heze Municipal People's Government Office | 2021/12/31 | Local (Municipal) |
| P33 | Notice on Issuing the "Qingdao City Long-Term Care Insurance Measures" | Qingdao Municipal People's Government | 2021/03/25 | Local (Municipal) |
| P34 | Opinions of the Shandong Provincial People's Government Office on Carrying out the Resident Long-Term Care Insurance Pilot Work | Shandong Provincial People's Government Office | 2022/11/10 | Local (Provincial) |
| P35 | Notice of the National Healthcare Security Administration Office on Issuing the "Long-Term Care Insurance Operational Procedures (Trial)" | National Healthcare Security Administration | 2024/09/23 | Central (National) |
| P36 | Notice of the National Healthcare Security Administration Office on Issuing the "Management Measures for Designated Long-Term Care Insurance Nursing Service Institutions (Trial)" | National Healthcare Security Administration | 2024/09/23 | Central (National) |
| P37 | Notice of the National Healthcare Security Administration Office on Issuing the "Model Service Agreement for Designated Long-Term Care Insurance Disability Assessment Institutions (Trial)" and "Model Service Agreement for Designated Long-Term Care Insurance Nursing Service Institutions (Trial)" | National Healthcare Security Administration | 2024/09/23 | Central (National) |
| P38 | Notice of the National Healthcare Security Administration Office on Issuing the "Interim Measures for the Management of the Long-Term Care Insurance Expert Database" | National Healthcare Security Administration | 2024/09/19 | Central (National) |
| P39 | Notice of the National Healthcare Security Administration Office on Issuing the "Coding Rules and Methods for Designated Service Institutions, Agreement Service Personnel, Entrusted Healthcare Security Service Agencies, and Staff of Entrusted Healthcare Security Service Agencies under Long-Term Care Insurance" | National Healthcare Security Administration | 2024/09/23 | Central (National) |
| P40 | Notice of the National Healthcare Security Administration on Issuing the "Management Measures for Designated Long-Term Care Insurance Disability Assessment Institutions (Trial)" | National Healthcare Security Administration | 2024/04/25 | Central (National) |
| P41 | Notice of the National Healthcare Security Administration and Ministry of Finance on Issuing the "Management Measures for Long-Term Care Insurance Disability Assessment (Trial)" | National Healthcare Security Administration, etc. | 2023/12/01 | Central (National) |
| P42 | Notice of the General Office of the Former China Banking and Insurance Regulatory Commission on "Launching the Pilot for Life Insurance and Long-Term Care Insurance Liability Conversion Business" | Former China Banking and Insurance Regulatory Commission | 2023/03/20 | Central (National) |
| P43 | Notice of the National Healthcare Security Administration Office and the General Office of the Ministry of Civil Affairs on Issuing the "Long-Term Care Disability Assessment Standards (Trial)" | National Healthcare Security Administration, etc. | 2021/07/16 | Central (National) |
| P44 | Notice of the General Office of the Former China Banking and Insurance Regulatory Commission on Regulating the Participation of Insurance Companies in the Long-Term Care Insurance System Pilot Services | Former China Banking and Insurance Regulatory Commission | 2021/05/25 | Central (National) |
| P45 | Guiding Opinions of the National Healthcare Security Administration and Ministry of Finance on Expanding the Long-Term Care Insurance System Pilot | National Healthcare Security Administration, etc. | 2020/09/10 | Central (National) |

**Table2 The scores of 45 policy texts**

| **Policy Code** | **X1** | **X2** | **X3** | **X4** | **X5** | **X6** | **X7** | **X8** | **X9** | **PMC Index** | **Depression Index** | **Policy Grade** | **Policy Level** |
| --- | --- | --- | --- | --- | --- | --- | --- | --- | --- | --- | --- | --- | --- |
| P1 | 0.80 | 0.67 | 0.67 | 0.50 | 1.00 | 0.50 | 0.67 | 1.00 | 1.00 | 6.80 | 2.20 | Acceptable | Local |
| P2 | 0.40 | 1.00 | 0.33 | 0.50 | 1.00 | 0.50 | 0.33 | 0.50 | 1.00 | 5.57 | 3.43 | Acceptable | Local |
| P3 | 0.40 | 1.00 | 0.33 | 0.50 | 0.40 | 0.50 | 0.00 | 0.67 | 1.00 | 4.80 | 4.20 | Poor | Local |
| P4 | 0.80 | 1.00 | 0.33 | 0.50 | 0.80 | 0.50 | 0.67 | 1.00 | 0.67 | 6.27 | 2.73 | Acceptable | Local |
| P5 | 1.00 | 1.00 | 0.33 | 0.50 | 1.00 | 0.50 | 0.33 | 1.00 | 1.00 | 6.67 | 2.33 | Acceptable | Local |
| P6 | 1.00 | 1.00 | 0.33 | 0.50 | 1.00 | 0.50 | 0.67 | 1.00 | 1.00 | 7.00 | 2.00 | Excellent | Local |
| P7 | 1.00 | 0.67 | 0.33 | 0.50 | 0.60 | 0.25 | 0.00 | 0.75 | 1.00 | 5.10 | 3.90 | Acceptable | Local |
| P8 | 0.40 | 1.00 | 0.33 | 0.50 | 0.60 | 0.50 | 0.00 | 1.00 | 1.00 | 5.33 | 3.67 | Acceptable | Local |
| P9 | 0.20 | 0.67 | 0.33 | 0.50 | 0.60 | 0.25 | 0.00 | 0.75 | 1.00 | 4.30 | 4.70 | Poor | Local |
| P10 | 0.80 | 0.67 | 0.67 | 0.50 | 1.00 | 0.75 | 0.67 | 0.75 | 1.00 | 6.80 | 2.20 | Acceptable | Local |
| P11 | 0.80 | 1.00 | 0.00 | 0.50 | 1.00 | 1.00 | 0.67 | 1.00 | 1.00 | 6.97 | 2.03 | Acceptable | Local |
| P12 | 1.00 | 0.67 | 0.33 | 0.50 | 1.00 | 0.75 | 0.67 | 1.00 | 1.00 | 6.92 | 2.08 | Acceptable | Local |
| P13 | 1.00 | 1.00 | 0.67 | 0.50 | 1.00 | 1.00 | 0.67 | 1.00 | 1.00 | 7.83 | 1.17 | Excellent | Local |
| P14 | 0.80 | 1.00 | 0.00 | 0.50 | 1.00 | 1.00 | 0.67 | 1.00 | 1.00 | 6.97 | 2.03 | Acceptable | Local |
| P15 | 0.80 | 1.00 | 0.00 | 0.50 | 1.00 | 1.00 | 0.33 | 1.00 | 1.00 | 6.63 | 2.37 | Acceptable | Local |
| P16 | 1.00 | 1.00 | 0.67 | 0.50 | 1.00 | 1.00 | 0.33 | 1.00 | 1.00 | 7.50 | 1.50 | Excellent | Local |
| P17 | 0.60 | 1.00 | 0.00 | 0.50 | 1.00 | 0.75 | 0.33 | 1.00 | 1.00 | 6.18 | 2.82 | Acceptable | Local |
| P18 | 0.80 | 1.00 | 0.33 | 0.50 | 1.00 | 1.00 | 0.33 | 0.75 | 1.00 | 6.72 | 2.28 | Acceptable | Local |
| P19 | 0.80 | 1.00 | 0.33 | 0.50 | 1.00 | 1.00 | 0.33 | 1.00 | 1.00 | 6.97 | 2.03 | Acceptable | Local |
| P20 | 0.80 | 1.00 | 0.33 | 0.50 | 1.00 | 1.00 | 0.67 | 0.75 | 1.00 | 7.05 | 1.95 | Excellent | Local |
| P21 | 0.60 | 1.00 | 0.33 | 0.50 | 0.40 | 0.75 | 0.00 | 0.75 | 1.00 | 5.33 | 3.67 | Acceptable | Local |
| P22 | 0.80 | 0.67 | 0.33 | 0.50 | 0.40 | 0.50 | 0.33 | 0.75 | 0.00 | 4.28 | 4.72 | Poor | Local |
| P23 | 0.80 | 0.33 | 0.33 | 0.50 | 1.00 | 0.00 | 0.33 | 0.00 | 0.00 | 3.30 | 5.70 | Poor | Local |
| P24 | 0.80 | 1.00 | 0.00 | 0.50 | 1.00 | 1.00 | 0.67 | 1.00 | 1.00 | 6.97 | 2.03 | Acceptable | Local |
| P25 | 1.00 | 1.00 | 0.33 | 0.50 | 1.00 | 1.00 | 0.67 | 1.00 | 1.00 | 7.50 | 1.50 | Excellent | Local |
| P26 | 1.00 | 1.00 | 0.33 | 0.50 | 1.00 | 1.00 | 0.67 | 1.00 | 1.00 | 7.50 | 1.50 | Excellent | Local |
| P27 | 0.80 | 1.00 | 0.00 | 0.50 | 1.00 | 0.75 | 0.33 | 0.75 | 0.67 | 5.80 | 3.20 | Acceptable | Local |
| P28 | 0.60 | 1.00 | 0.00 | 0.50 | 0.20 | 0.75 | 0.00 | 0.50 | 1.00 | 4.55 | 4.45 | Poor | Local |
| P29 | 0.80 | 1.00 | 0.33 | 0.50 | 1.00 | 1.00 | 0.33 | 0.75 | 1.00 | 6.72 | 2.28 | Acceptable | Local |
| P30 | 0.80 | 1.00 | 0.33 | 0.50 | 1.00 | 1.00 | 0.67 | 0.75 | 1.00 | 7.05 | 1.95 | Excellent | Local |
| P31 | 1.00 | 1.00 | 0.33 | 0.50 | 1.00 | 0.50 | 0.33 | 1.00 | 0.67 | 6.33 | 2.67 | Acceptable | Local |
| P32 | 0.60 | 1.00 | 0.33 | 0.50 | 0.40 | 0.00 | 0.33 | 0.75 | 0.00 | 3.91 | 5.09 | Poor | Local |
| P33 | 0.80 | 1.00 | 0.33 | 0.50 | 1.00 | 1.00 | 0.67 | 1.00 | 1.00 | 7.30 | 1.70 | Excellent | Local |
| P34 | 1.00 | 1.00 | 0.33 | 0.50 | 0.80 | 0.50 | 0.33 | 1.00 | 0.67 | 6.13 | 2.87 | Acceptable | Local |
| P35 | 0.80 | 1.00 | 0.33 | 0.50 | 0.60 | 0.50 | 0.33 | 0.50 | 1.00 | 5.56 | 3.44 | Acceptable | Central |
| P36 | 0.80 | 0.67 | 0.33 | 0.50 | 0.40 | 0.75 | 0.33 | 0.50 | 1.00 | 5.28 | 3.72 | Acceptable | Central |
| P37 | 0.60 | 0.67 | 0.33 | 0.50 | 0.60 | 0.75 | 0.33 | 0.50 | 0.67 | 4.95 | 4.05 | Poor | Central |
| P38 | 0.80 | 0.67 | 0.67 | 0.50 | 0.60 | 0.75 | 0.33 | 0.75 | 1.00 | 6.07 | 2.93 | Acceptable | Central |
| P39 | 0.60 | 0.67 | 0.33 | 0.50 | 0.20 | 0.75 | 0.33 | 0.50 | 0.33 | 4.21 | 4.79 | Poor | Central |
| P40 | 1.00 | 1.00 | 0.33 | 0.50 | 0.40 | 0.75 | 0.33 | 0.75 | 0.67 | 5.73 | 3.27 | Acceptable | Central |
| P41 | 0.80 | 1.00 | 0.67 | 0.50 | 0.60 | 0.75 | 0.33 | 0.75 | 1.00 | 6.40 | 2.60 | Acceptable | Central |
| P42 | 0.80 | 1.00 | 0.33 | 0.50 | 0.60 | 0.75 | 0.33 | 1.00 | 0.33 | 5.64 | 3.36 | Acceptable | Central |
| P43 | 0.60 | 0.67 | 0.67 | 0.50 | 0.80 | 0.50 | 0.33 | 1.00 | 0.67 | 5.74 | 3.26 | Acceptable | Central |
| P44 | 1.00 | 0.67 | 0.33 | 0.50 | 0.20 | 0.50 | 0.33 | 1.00 | 0.33 | 4.86 | 4.14 | Poor | Central |
| P45 | 0.80 | 0.67 | 0.33 | 0.50 | 0.60 | 0.50 | 0.33 | 0.75 | 0.67 | 5.15 | 3.85 | Acceptable | Central |
| Mean ± Std | 0.78±0.19 | 0.89±0.17 | 0.33±0.19 | 0.5±0 | 0.77±0.28 | 0.69±0.27 | 0.39±0.22 | 0.82±0.22 | 0.83±0.3 | 6.01±1.1 | 2.99±1.1 | Acceptable | — |
